# Marsupial Genome Sheds Light on the Evolution of Immunity

**DOI:** 10.1371/journal.pbio.0040075

**Published:** 2006-01-31

**Authors:** Emma Hill

All organisms need defense systems to ward off infection. These defenses typically mobilize in response to antigens, bits of protein fragments derived from a pathogen. Vertebrates have a superbly efficient system of adaptive immunity that uses proteins to recognize and bind billions of different antigens via a process known as antigen presentation. Ultimately, this process leads to removal of the antigen. Antigen binding proteins are encoded within one large genomic region known as the major histocompatibility complex (MHC). The high level of polymorphism, or variation, in this genomic region allows the adaptive immune system to keep pace with an ever-changing array of antigens.

Genes in the MHC region are subdivided into Class I, Class II, and Class III based on their structure and function. The organization of MHC genes in present-day species can provide insight into how immunity has evolved. To date, maps of the MHC are available from eutherian (placental) mammals and nonmammalian species, including birds, bony fish, and cartilaginous fish such as sharks. But MHC organization and complexity differ substantially between mammals and nonmammals, making it difficult to infer the region's evolutionary history, or phylogeny.

Using available marsupial opossum *(Monodelphis domestica)* genome sequences, Katherine Belov, Robert Miller, and their colleagues present a detailed map of the opossum MHC. These data from a noneutherian mammal help bridge the gap between nonmammals and eutherian mammals, to enable a more comprehensive phylogeny of the MHC.

In eutherian mammals, the MHC is large and dense, with 264 genes and pseudogenes (remnants of once functional genes) scattered over 3.6 Mb and ordered as Class I–III–II in adjacent gene blocks in humans. In nonmammals, the MHC has fewer genes (the chicken has only 19 genes over 92 kb) ordered as Class III–I–II. The authors found that the MHC in the opossum is flanked at either end by the same marker genes that border the human MHC. This noneutherian mammalian MHC spans 3.95 Mb and encodes 114 genes (identified via sequence similarity to genes from other species), sharing 87 with the human MHC. The authors have provided a Web-based genome browser (http://bioinf.wehi.edu.au/opossum) for viewing an annotated map of the opossum MHC region.

Belov et al. found that while the size and complexity of the opossum MHC is closer to eutherian mammals, its organization is closer to fish and birds: in the opossum, Class I genes are interspersed with the Class II genes—this has not been seen in any other animals. This similar grouping of Class I and II genes implies that they may have been arranged this way in the mammalian ancestor (supported also by the presence of the Class I pseudogenes in the human Class II region).

Humans and mice have a conserved framework region, consisting of a set of genes that are interspersed among the Class I loci. The opossum also contains these genes next to the Class III region. The authors deduced that this framework region assembled in the ancestral mammalian MHC before Class I genes moved to this location in eutherians.

Only one of the opossum MHC genes, *UA1*, has the characteristics of what's known as a classical Class I gene—high polymorphism and ubiquitous expression. The authors saw transcripts of this gene in all tissues tested. Expression of a single classical Class I gene is unusual in mammals but has been seen in the chicken and the frog. Contrary to a previous hypothesis that Class I gene evolution is constrained by their proximity to antigen processing genes, the Class I genes in the opossum are significantly diverged.

The authors identified three marsupial-specific classical Class II gene families (*DA*, *DB*, and a newly discovered family designated *DC*). They also found that sequences responsible for regulating Class I genes—SXY motifs—known to be important in the eutherian MHC, have diverged in the opossum. Conversely, the Class II SXY regions are quite well conserved. This is logical given that Class I genes are more likely to evolve new functions while Class II genes are typically coexpressed.

Significantly, the authors also found data supporting the idea that there was an ancient relationship between the MHC and another critical component of the immune system, the natural killer complex (NKC), which contains natural killer (NK) cell receptor loci. Two NKC genes, *OSCAR* and *MIC*, are present in the opossum MHC. As MHC Class I molecules are ligands (activators) for NK cell receptors, it makes sense that these two gene families coevolve.

The authors conclude that vertebrates once possessed an “immune supercomplex” that performed the MHC functions. While this complex no longer exists in modern genomes, scientists can probe its remnant traces for clues to its history. Gaining a better understanding of how our mammalian immunity evolved will lead to a much broader appreciation of how organisms' internal defense systems work across a wide range of species.

## 

**Figure:**
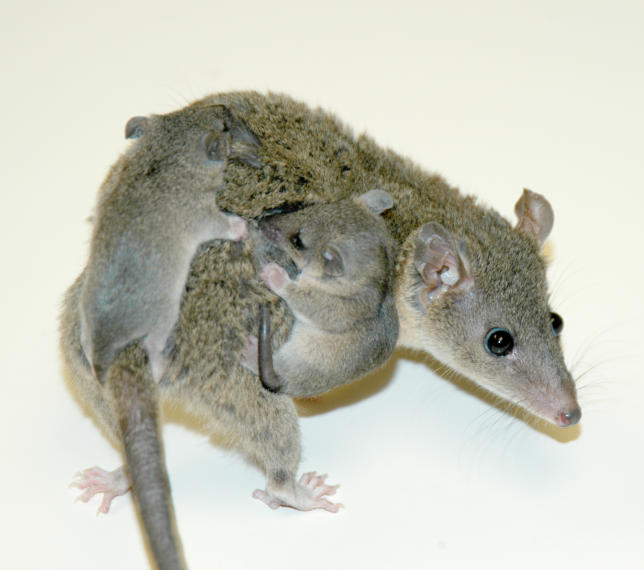
Genome sequence data from the opossum fills a gap in our understanding of the evolution of the mammalian immune system.

